# Impact of Cafeteria Service Discontinuation at a Dialysis Facility on Medium-Term Nutritional Status of Elderly Patients Undergoing Hemodialysis

**DOI:** 10.3390/nu14081628

**Published:** 2022-04-14

**Authors:** Satoko Notomi, Mineaki Kitamura, Kosei Yamaguchi, Takashi Harada, Tomoya Nishino, Satoshi Funakoshi, Kazue Kuno

**Affiliations:** 1 Graduate School of Human Care Sciences, Nishikyushu University, Saga 842-8585, Japan; s-notomi@nagajin.jp (S.N.); kunok@me.com (K.K.); 2 Nagasaki Renal Center, Nagasaki 850-0032, Japan; tugumasayamaguchi@gmail.com (K.Y.); renaltharada@nagajin.jp (T.H.); satoshi2754@yahoo.co.jp (S.F.); 3 Department of Nephrology, Nagasaki University Graduate School of Biomedical Sciences, Nagasaki 852-8523, Japan; tnishino@nagasaki-u.ac.jp

**Keywords:** elderly patients, hemodialysis, malnutrition, eating habits, cafeteria services, COVID-19 measures

## Abstract

Despite evident lifestyle changes due to measures against the coronavirus disease 2019 (COVID-19) outbreak, few reports focus on the effects of eating-behavior changes on the nutritional status of elderly patients undergoing hemodialysis (HD). Thus, we examined dry-weight reduction, the simplest indicator of malnutrition among patients undergoing dialysis, and its association with the discontinuation of cafeteria services at a dialysis facility as per COVID-19 measures. This retrospective study included elderly patients (aged ≥ 65 years) undergoing HD at the Nagasaki Renal Center between December 2020 and October 2021. We collected nutrition-related data and patient characteristics and evaluated the association between the service discontinuation and dry-weight reduction 10 months after the discontinuation using multivariable logistic regression. This study included 204 patients, 79 of which were cafeteria users. During the observation period, cafeteria users showed significant dry-weight reduction; however, this was not observed among non-users. Multivariable logistic regression analysis indicated a close association between dry-weight reduction and the service discontinuation. That is, the dietary services cancelation caused dry-weight reduction in patients who relied on the cafeteria. As elderly patients undergoing HD are vulnerable to changes in their eating environment, alternative nutritional management methods need to be considered.

## 1. Introduction

The ongoing coronavirus disease 2019 (COVID-19) pandemic and related public health measures are likely to negatively affect lifestyle behavior [1]. Recent studies have shown decreased physical activity in various populations, particularly in older adults [2,3]. Regarding eating behavior, a recent study reported older adults’ tendency to reduce food intake and lose body weight during the pandemic [4].

In preventing the spread of infection [5], it has become difficult for many people to eat meals with a large number of people. This is even more true for patients undergoing hemodialysis (HD) as they have an increased risk of severe COVID-19 infection due to immune deficiency [6]; therefore, careful attention should be paid to how safely meals are served in dialysis facilities. The Nagasaki Renal Center is a specialized HD facility providing cafeteria services for patients undergoing HD. As HD treatment takes 3–6 h, their lunch or dinner time should overlap their treatment. Our cafeteria service compensated for malnutrition in patients undergoing HD, particularly in those with irregular eating habits. Additionally, older adult patients tend to have difficulty preparing a desirable dialysis diet on their own because they consider activities of daily living, such as shopping and cooking, a burden [7].

Our facility continued the cafeteria service, taking measures against COVID-19 as per the instructions of the healthcare facility in Japan. However, because the number of patients with COVID-19 in Japan began to increase from mid-December 2020, under government policies, the cafeteria services were unavoidably canceled on 18 December 2020. Furthermore, these services are unlikely to resume until the pandemic is contained.

Under such circumstances, we hypothesized that the discontinuation of cafeteria services could change patients’ eating behaviors and cause malnutrition. Here, we examined the association between dry-weight reduction and the use of cafeteria services to determine the effect of ceasing such services. Dry weight is the target body weight at the end of dialysis treatment (patients need fluid removal during HD). Patients can maintain normal blood pressure until their next HD. Dry weight varies over time as lean body mass and body fat change [8]. Dry-weight reduction is the simplest indicator of malnutrition at dialysis facilities and a decisive prognostic factor [9].

We also hypothesized that elderly patients are more vulnerable to the changes in their eating environment, based on several studies showing that older adults are more susceptible to the changes related to the COVID-19 pandemic than younger adults [10,11,12,13]. However, few studies have examined the effects of changes in older adults’ eating behavior and nutritional status during the COVID-19 outbreak [4,14]. Furthermore, no studies have examined the changes in eating behavior caused by COVID-19 in elderly patients undergoing HD who are susceptible to malnutrition [15].

This study aims to (i) elucidate the medium-term dry-weight reduction of elderly patients undergoing HD under the influence of the discontinuation of cafeteria services and (ii) clarify changes in their eating behavior due to this discontinuation.

## 2. Materials and Methods

### 2.1. Patient Background

**Figure 1:**
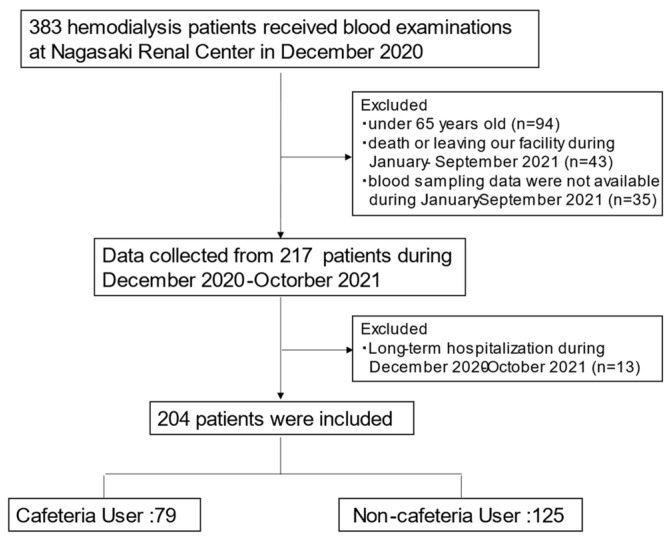
Patient flowchart.

The subjects were elderly (aged ≥ 65 years) patients undergoing HD who were continuously treated at the Nagasaki Renal Center between December 2020 and October 2021. Patients who left the Nagasaki Renal Center or died during the observation period were excluded. Patients who did not undergo routine blood examinations were also excluded. Figure 1 shows our patient flow chart.

### 2.2. Data Collection

Patient characteristics such as age, sex, duration of dialysis, dry weight, body mass index (BMI), blood examinations, complications, and living conditions were obtained from medical records during the observation period. Regarding blood examinations, nutrition-related items, such as cholinesterase (ChE), total protein (TP), total cholesterol (TC), triglycerides (TG), creatinine (Cr), potassium, phosphorus, hemoglobin (Hb), hematocrit (Ht), intact parathyroid hormone (iPTH), normalized protein catabolic rate (nPCR), albumin (Alb), and geriatric nutritional risk index (GNRI), were extracted from regular blood examinations at our facility. The GNRI was calculated based on the patient’s serum albumin and body weight using the modified version proposed by Yamada et al. [16,17]; GNRI = (14.89 × albumin [g/dL]) + [41.7× (bodyweight/ideal body weight); if body mass index (BMI) exceeded 22, bodyweight/ideal body = 1].

Nutrition-related items were obtained in December 2020 as the baseline and one month (January 2021), four months (April 2021), seven months (July 2021), and ten months (October 2021) after stopping the service.

### 2.3. Statistical Analyses

Categorical values are shown as numbers (%), and continuous variables as mean ± standard deviation. Non-normally distributed data were presented as median values with interquartile ranges. The participants were divided into two groups depending on whether their dry weight had decreased between December 2020 and October 2021. The chi-square test or Wilcoxon sum test was used to compare the two groups. Logistic regression analysis was performed to elucidate the association between dry-weight reduction and patient background. Additionally, a multivariable logistic regression analysis was performed, including parameters with *p* values less than 0.05 in the univariable logistic regression analysis. Changes in parameters related to energy intake or nutritional status (TG, TP, nPCR, GNRI, BUN, Cr, and CRP levels) within the same group were verified using the Wilcoxon signed-rank test. A comparison of the decrease from baseline in the parameters between cafeteria and non-cafeteria users was performed using the Wilcoxon rank-sum test. Statistical significance was set at *p* < 0.05. Regarding multiple comparisons in the same group, Bonferroni correlation was used, and a *p*-value < 0.05/4 = 0.0125 was considered statistically significant. Statistical analyses were performed using JMP 15 software (SAS Institute Inc., Cary, NC, USA).

## 3. Results

### 3.1. Patient Background

In December 2020, when the cafeteria services were canceled, 383 patients underwent HD at the Nagasaki Renal Center. After excluding 179 patients, 204 patients (74.8 ± 7.7 years; 54.0% males) were analyzed. Among them, 79 were cafeteria users and 125 were non-cafeteria users (Figure 1). Patient characteristics are shown in Table 1. Except for the percentage of nursing-home residents and serum calcium levels, there were no significant differences between the cafeteria and non-cafeteria users.

### 3.2. Dry-Weight Reduction and Changes in Parameters by Use of Cafeteria Services

Univariable logistic regression analysis for dry-weight reduction was conducted based on the patients’ backgrounds. Age, dialysis time, cafeteria use, BMI, ChE, BUN, Cr, and CRP levels were statistically significant (Table 2). Next, we conducted a multivariable logistic regression model including these parameters; patients classified by dry-weight reduction were associated with cafeteria use, CRP, age, and BUN (*p* < 0.001, 0.005, 0.009, and 0.028, respectively) (Table 2).

**Figure 2:**
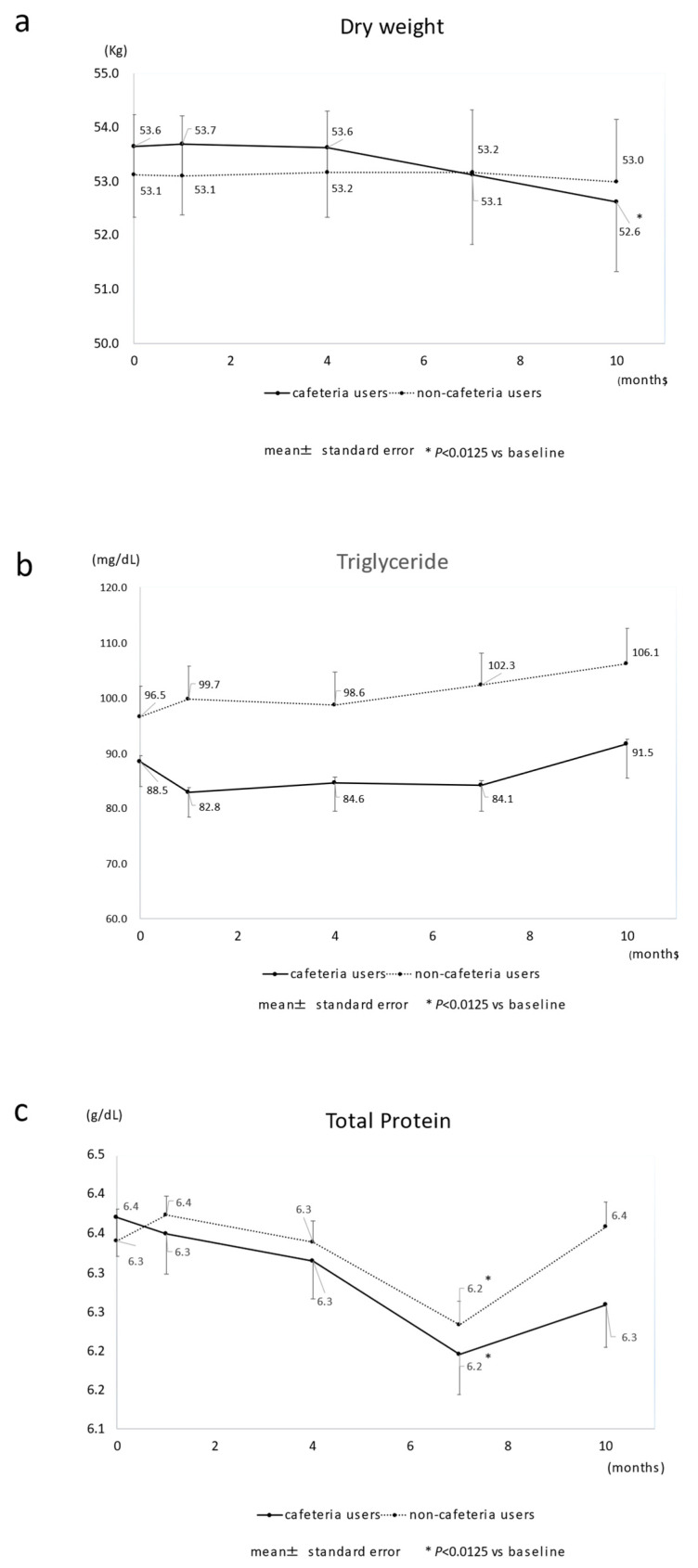

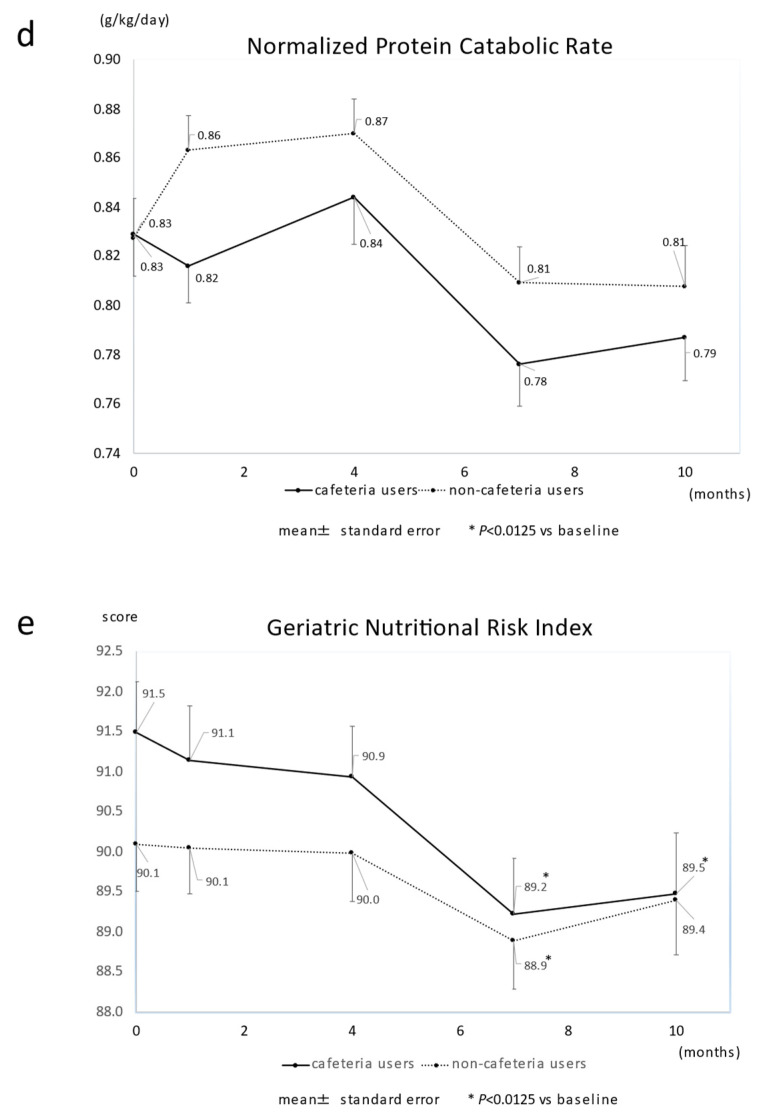
Changes in major nutrition-related parameters in cafeteria users and non-cafeteria users. The cafeteria closed at time 0. (**a**) dry weight; (**b**) triglyceride; (**c**) total protein; (**d**) normalized protein catabolic rate; (**e**) geriatric nutrition risk index. * *p* < 0.0125 vs. baseline.

Changes in the parameters (TG, TP, nPCR, GNRI, BUN, Cr, and CRP levels) within the same group are shown in Figure 2a–e and Supplementary Table S1. Comparisons of the decrease from baseline between the two groups are shown in Supplementary Table S2.

Regarding the changes within the same group (Supplementary Table S1), the cafeteria users’ dry weight decreased significantly 7 and 10 months after discontinuing the services, but no significant decrease was seen in non-cafeteria users. The TP, nPCR, and GNRI levels tended to decline over time among cafeteria users.

**Figure 3:**
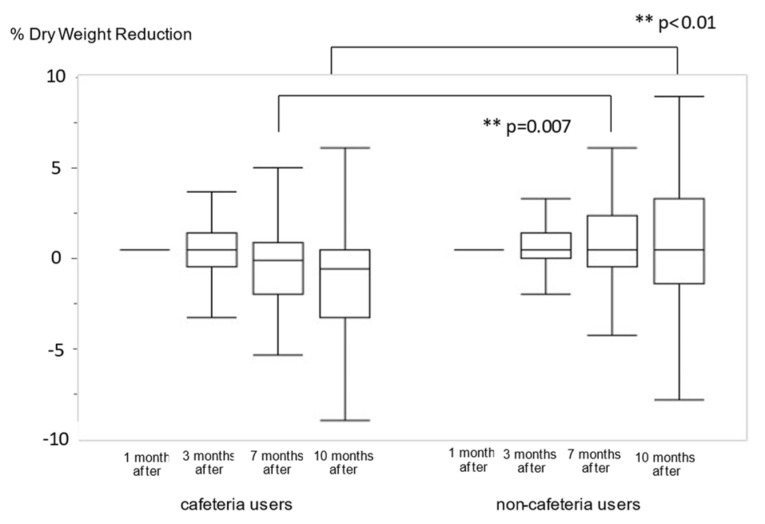
The comparison of percentage changes in dry-weight reduction between cafeteria users and non-cafeteria users. ** *p* < 0.01 vs. non-cafeteria users.

There was no change in the median weight loss rate among non-cafeteria users, but the median of the percent change in dry-weight reduction was 0.8% after 7 months and 1.2% after 10 months among cafeteria users. There was a significant difference between the two groups after 7 (*p* = 0.007) and 10 months (*p* < 0.001) (Figure 3 and Supplementary Table S2). For the TG level, significant differences were observed throughout the observation period. The TP and GNRI levels deteriorated over time; there was a significant difference after 10 months (Supplementary Table S2).

## 4. Discussion

After the cafeteria services discontinuation as per COVID-19 health measures, we investigated the changes in dry weight and nutrition-related items in elderly patients receiving HD in a single center. Among our participants, dry-weight reduction was associated with the use of cafeterias, CRP, age, and BUN. In the 7–10 months after the cancelation of the cafeteria service, dry-weight reduction was significantly greater among cafeteria users, indicating that nutritional status had deteriorated over a long period.

Globally, malnutrition in patients undergoing HD is widespread (28%–54%) [18,19] with a greater risk of mortality [18,19,20]. In patients undergoing HD, uremic toxins such as indoxyl sulfate evoke oxidative stress and insulin resistance, resulting in a state called protein-energy wasting (PEW) [21,22]. As aging progresses, the problem of malnutrition is highlighted, and PEW in elderly HD patients has become an inevitable concern [23]. We focused on dry-weight reduction, which is the simplest indicator of malnutrition at dialysis facilities and one of the PEW diagnosis criteria [24]. In this study, several factors affecting dry-weight reduction were shown, the biggest of which was the discontinuation of cafeteria services (Table 2).

Since weight loss is caused by energy shortage, cafeteria users were thought to lack suitable energy intake after the services were ceased. There are three possible reasons for this shortage in energy intake.

First is the difficulty in self-managing their dialysis-related diet. According to a diet review of dialysis patients, the adherence to the target energy intake was 23.1%, protein, 45.5%, and fat 41.4% [25], indicating that the biggest problem with the patients’ diet is their low energy-intake. To compensate for malnutrition status, the cafeteria provided the ideal meals based on the dietary standards of dialysis patients in Japan (energy 605 kcal, protein 20.1 g, fat 17.0 g, carbohydrates 88.6 g: average measured value for November 2020). It is probable that the difficulties elderly patients have in self-managing their energy intake led to decreased energy intake after the services were discontinued.

Second is the changes in their mealtimes. The cafeteria users had lunch immediately after dialysis. After the services were discontinued, they had to take lunch at home; that is, they took their lunch later than before. Therefore, they may have lost the opportunity to have dinner in the late afternoon. Patients who relied on the cafeteria for their dinner before starting HD did not have enough time for dinner after work.

Third, “frailty” is a characteristic of the elderly. It is a state of vulnerability to poor homeostasis resolution after a stressor event [26]. Quarantine changes eating habits, expands social isolation—a stressor for the older adults [27]—and promotes frailty. The onset of frailness is explained by the theory of “frail cycle,” a vicious cycle in which insufficient food intake leads to weight loss, malnutrition, and decreased activity, leading to a further decrease in food intake [28]. The cafeteria users had difficulties adapting to changes in their eating habits caused by the discontinuation of the cafeteria, and this stressor accelerated their frailty and resulted in their dry-weight reduction. In this study, age was the second largest factor associated with dry-weight reduction (Table 2), indicating that elderly patients are more vulnerable to stress and are more likely to fall into frailty. According to a previous review, living alone can be one of the socioeconomic factors that lead to malnutrition in older adults [29]; however, it was not associated with dry-weight reduction (Table 2). In our participants, changes in the eating environment may be a more serious factor affecting malnutrition than in the original living conditions.

One of the important results of this study was that dry-weight reduction occurred slowly among cafeteria users. Compared to the PEW diagnostic criteria (5% of weight loss at 3 months and 10% at 6 months) [24], the dry-weight reduction rate was less than a quarter (0.8% at 7 months and 1.2% at 10 months; Figure 3 and Supplementary Table S2). Therefore, the effect of the cafeteria’s discontinuation has been overlooked by both the patients and medical staff. However, this study revealed that the discontinuation of the cafeteria had a significant impact on medium-term dry-weight reduction in elderly patients undergoing HD. Dry weight is adjusted by clinical signs, such as blood pressure and cardiothoracic ratio (CTR) diagnosed by X-ray [8]; therefore, dry-weight data obtained from medical records may have taken some time to reflect the actual lean body mass. This means that substantial weight-loss in cafeteria users could have occurred earlier than dry-weight reduction. Notably, cafeteria users unexpectedly fell into malnutrition under the COVID-19 measures, making it crucial to consider improving their nutritional status.

CRP level was also a factor that affected dry-weight reduction (Table 2). PEW is characterized as malnutrition associated with inflammation [30], and several studies have shown a strong association between malnutrition and inflammation in patients undergoing HD [31,32,33]. In this study, there was no consecutive significant increase in CRP levels among users (Supplementary Table S1). However, it is plausible that dry-weight reduction is more likely to occur in patients with inflammation. BUN levels were also associated with dry-weight reduction (Table 2). BUN levels tend to be low in patients with low dietary intake [34]. A previous study showed that older adult people who eat less are at greater risk of losing weight under COVID-19 prevention measures [5]; this phenomenon was also observed in this study.

In addition to the reduction in dry weight, we also examined changes in parameters related to energy intake or nutritional status (TG, TP, nPCR, GNRI, BUN, and Cr levels). Cafeteria users’ TG levels did not increase as much as those observed in non-users (Figure 2b and Supplementary Table S1). As TG levels showed the earliest decrease among the parameters (Supplementary Table S2), TG levels can be a sensitive indicator of an energy shortage. nPCR, used as a surrogate for daily dietary protein intake [35], tended to decrease further in cafeteria users over time (Figure 2d and Supplementary Table S1). Inconsistent with the reduced energy intake, protein intake may have decreased in users. TP levels decreased significantly in users after 7 months (Supplementary Table S1). When adipose tissue is depleted, as energy intake decreases, the body uses proteins for energy [36]. TG levels decreased soon after discontinuing the service, whereas a drop in TP level occurred 7 months after (Supplementary Table S2). This discrepancy can be explained by the protein catabolism increasing to compensate for the energy shortage. The GNRI, which indicates the nutritional status of patients on HD, deteriorated over time for both users and non-users. However, the decrease was larger in cafeteria users (Figure 2e and Supplementary Table S2). Dry-weight reduction accelerates the aggravation of GNRI in cafeteria users. There was no significant difference in BUN and Cr between the groups (Supplementary Table S2). These two indicators are affected not only by malnutrition but also by dialysis conditions and other factors, such as amino-acid loss during HD [21,37,38,39]. Therefore, it was difficult to clarify the effect of the cafeteria’s discontinuation on these indicators in this study.

This study revealed that changes in eating behavior tend to be stressors that lead to weight loss in elderly patients undergoing HD. To prevent the vicious circle of frailty, early detection and nutritional intervention are crucial [40]. The TG level decreased soon after cafeteria services ceased, probably due to energy shortage. Contrastingly, early appropriate energy supply to those with low TG could be an effective nutritional intervention. As we cannot predict the end of COVID-19 at this time, there is no prospect of reopening the cafeteria. Nonetheless, the nutritional status gradually deteriorated in our participants, and there is a concern that long-lasting malnutrition will affect their prognoses. To improve nutritional management, meal-delivery services that can supply appropriate energy should be considered.

This study has several limitations. First, this study was conducted at a single center; the reliability of the study data was not guaranteed, and the results may have been influenced by participant characteristics. For example, nursing-home residents were included in this study, more than half of which lived in a nursing home associated with our hospital and located nearby. Therefore, their nutritional characteristics may differ because they were assured meals three times a day at their facilities. These differences may have affected our results. Second, because we excluded patients who died during the observation period, the results may have become skewed. Third, as this is a retrospective study based on clinical practice, we mainly used data from medical records. We used regular blood-sampling results and did not collect high-sensitivity CRP levels as indicators of inflammation. Highly sensitive CRP levels are needed to examine the relationship between malnutrition and inflammation precisely. Moreover, we could not obtain our participants’ detailed dietary records, including size or calorific value records. This information is needed to elucidate the precise impact of cafeteria discontinuation.

## 5. Conclusions

The discontinuation of cafeteria services at a dialysis facility during the COVID-19 pandemic affects the medium-term nutritional status of elderly patients undergoing HD. As patients undergoing HD are vulnerable to malnutrition, it is crucial to consider alternative nutritional management methods to cafeteria services, even before COVID-19 is endemic.

## Figures and Tables

**Table 1:** Summary of the patient background.

	Cafeteria Users (*n* = 79)	Non-Cafeteria Users (*n* = 125)	*p* Value
Age (years)	73.0 (68.0–80.0)	72.0 (69.0–80.5)	0.70
Male (%)	46 (58.2)	65 (52.0)	0.38
Dialysis vintage (month)	66 (28–102)	51 (22–120)	0.43
Dialysis time (hour)	4.0 (3.5–4.0)	4.0 (3.5–4.0)	0.35
DM history (%)	16 (20.3)	36 (28.8)	0.19
Dementia (%)	6 (7.6)	18 (14.4)	0.18
Living alone (%)	18 (22.8)	20 (16.0)	0.27
Nursing-home residents (%)	1 (1.3)	18 (14.4)	0.0018
Dry weight (kg)	53.0 (44.0–61.0)	53.2 (42.8–61.0)	0.76
Body mass index (kg/m^2^)	21.1 (18.1–23.8)	20.7 (18.8–22.6)	0.82
ChE (U/L)	192 (167–223)	210 (165–239)	0.23
TP (g/dL)	6.4 (6.1–6.7)	6.4 (6.1–6.6)	0.78
AST (U/L)	15 (11–18)	14 (11–16)	0.15
ALT (U/L)	10 (7–13)	9 (6–12)	0.29
CK (U/L)	71 (47–93)	60 (39–85)	0.07
TC (mg/dL)	149 (127–180)	150 (126–176)	0.93
TG (mg/dL)	84 (55–116)	85 (56–111)	0.89
cCa (mg/dL)	8.7 (8.3–9.0)	8.5 (8.1–8.8)	0.0075
UA (mg/dL)	6.6 (5.6–7.5)	6.9 (6.1–7.9)	0.07
BUN (mg/dL)	57.8 (48.8–67.4)	60.5 (51.8–70.3)	0.14
Cr (mg/dL)	9.26 (7.92–10.45)	8.97 (7.26–10.57)	0.28
K (mEq/L)	4.7 (4.2–5.1)	4.7 (4.1–5.2)	0.95
P (mg/dL)	5.1 (4.5–6.0)	5.3 (4.5–6.1)	0.46
CRP (mg/dL)	0.16 (0.08–0.61)	0.2 (0.08–0.49)	0.87
Hb (g/dL)	11.0 (10.2–11.8)	10.7 (10.0–11.5)	0.19
Ht (%)	34.5 (31.1–36.0)	33.2 (31.1–35.4)	0.16
Alb (g/dL)	3.6 (3.4–3.7)	3.5 (3.3–3.8)	0.10
GNRI	91.3 (88.2–95.7)	90.8 (85.3–95.0)	0.24
nPCR (g/kg/day)	0.80 (0.72–0.92)	0.82 (0.69–0.94)	0.77
Intact PTH (pg/mL)	93 (28–143)	72 (37–134)	0.83
KT/V	1.6 (1.3–1.9)	1.5 (1.2–1.8)	0.06

Chi-square test and Wilcoxon sum test were used for analysis. Dialysis vintage—dialysis treatment period at the start of the observation period; DM—diabetes mellitus—ChE—Cholinesterase; TP—Total Protein; AST—aspartate aminotransferase; ALT—alanine aminotransferase; CK—creatine kinase; TC—total cholesterol; TG—triglycerides; cCa—corrected calcium; UA—uric acid; BUN—blood urea nitrogen; Cr—creatinine; K. potassium; P—phosphate; CRP—c-reactive protein; Hb—hemoglobin; Ht—hematocrit; Alb—albumin; GNRI—geriatric nutritional risk index; nPCR—normalized protein catabolism rate; PTH—parathyroid hormone.

**Table 2:** Logistic regression models for dry-weight reduction.

	Univariate			Multivariable		
	OR	95% CI	*p* Value	OR	95% CI	*p* Value
Age per 1 year old	1.08	1.04–1.13	<0.001	1.07	1.01–1.19	0.009
Male vs. Female	1.01	0.58–1.76	0.97			
Dialysis vintage per 1 month	0.99	0.96–1.03	0.74			
Dialysis time per 1 hr	0.50	0.28–0.86	0.01	0.69	0.37–1.29	0.23
DM history	1.64	0.86–3.10	0.15			
Dementia	1.13	0.48–2.70	0.78			
Cafeteria users	2.62	1.47–4.74	0.001	3.23	1.66–6.28	<0.001
Living Alone	1.22	0.60–2.50	0.57			
Nursing-home residents	0.50	0.17–1.34	0.17			
Dry weight per 1 kg (kg)	0.98	0.95–1.00	0.06			
Body mass index per 1 kg/m^2^	0.92	0.85–0.99	0.037	0.96	0.88–1.06	0.44
ChE per 1 unit U/L	0.94	0.89–0.99	0.016	0.99	0.93–1.05	0.65
TP per 1 g/dL	1.23	0.66–2.48	0.47			
AST per 1 U/L	1.00	0.97–1.04	0.88			
ALT per 1 U/L	0.99	0.95–1.02	0.54			
TC per 1 mg dL	1.00	0.99–1.01	0.60			
TG per 1 mg/dL	0.95	0.90–1.00	0.08			
cCa per 1 mg/dL	1.03	0.61–1.75	0.91			
UA per 1 mg/dL	0.88	0.73–1.06	0.18			
BUN per 1 mg/dL	0.72	0.56–0.89	0.002	0.77	0.60–0.98	0.028
Cr per 1 mg/dL	0.82	0.72–0.93	0.002	0.93	0.79–1.10	0.41
CRP per 1 mg/dL	1.56	1.09–2.53	0.011	1.79	1.08–2.98	0.005
Hb per 1 g/dL	0.92	0.71–1.20	0.55			
Alb per 1 g/dL	0.49	0.18–1.24	0.13			
KT/V per 1 Unit	0.54	0.57–1.00	0.053			

OR—odds ratio; CI—confidence interval; ChE—cholinesterase; TP—total protein; AST—aspartate aminotransferase; ALT—alanine aminotransferase; TC—total cholesterol; TG—triglycerides; cCa—corrected calcium; UA—uric acid; BUN—blood urea nitrogen; Cr—creatinine; CRP—c-reactive protein; Hb—hemoglobin; Alb—albumin.

## Data Availability

The datasets analyzed in the current study are available from the corresponding author upon reasonable request.

## Supplementary Materials

The following are available online at https://www.mdpi.com/article/10.3390/nu14081628/s1, Table S1: Changes in parameters within the same group. Table S2: Comparison of the decrease in parameters between cafeteria users and non-cafeteria users
