# Supplementary material for: Impact of Cafeteria Service Discontinuation at a Dialysis Facility on Medium-Term Nutritional Status of Elderly Patients Undergoing Hemodialysis
Source: Nutrients. 2022 Apr 14;14(8):1628. doi: 10.3390/nu14081628 (PMC9031672; doi:10.3390/nu14081628)
Supplement: Supplementary file 1 [file nutrients-14-01628-s001.zip › nutrients-1645510-supplementary.pdf]

Supplementary Table S1. Changes in parameters within the same group

		0 month	1 month after	*P*	4 months after	*P*	7 months after	*P*	10 months after	*P*
DW (kg)	users	53.0 (44.0-61.0)	52.5 (44.0-61.5)	0.48	52.5 (44.0-61.0)	0.91	52.8 (43.5-61.0)	0.02	52.0 (43.5-61.5)	<0.001
	non- users	53.2 (42.8-61.0)	53.2 (43.0-60.8)	0.73	53.0 (43.0-60.7)	0.65	53.2 (42.8-60.7)	0.77	52.5 (42.5-61.0)	0.53
TG (mg/dL)	users	84 (55-116)	74 (54-99)	0.07	77 (53-103)	0.37	75 (55-105)	0.30	82 (54-110)	0.52
	non- users	85 (56-111)	85 (58-122)	0.24	80 (57-113)	0.56	85 (60-125)	0.09	84 (63-127)	0.02
TP (g/dL)	users	6.4 (6.1-6.7)	6.3 (6.1-6.6)	0.47	6.3 (6.1-6.6)	0.10	6.3 (5.9-6.5)	<0.001	6.3 (5.9-6.6)	0.006
	non- users	6.4 (6.1-6.6)	6.4 (6.1-6.6)	0.15	6.3 (6.1-6.6)	0.93	6.2 (6.0-6.5)	<0.001	6.4 (6.0-6.6)	0.55
nPCR (g/kg/day)	users	0.80 (0.72-0.92)	0.82 (0.74-0.91)	0.38	0.82 (0.74-0.94)	0.41	0.79 (0.70-0.86)	0.01	0.77 (0.69-0.87)	0.03
	non- users	0.82 (0.69-0.94)	0.85 (0.76-0.98)	0.003	0.87 (0.75-0.96)	0.004	0.79 (0.69-0.93)	0.22	0.79 (0.67-0.93)	0.24
GNRI	users	91.3 (88.2-95.7)	91.4 (86.8-95.9)	0.21	91.4 (86.7-95.3)	0.08	89.3 (84.9-93.8)	<0.001	90.4 (84.3-94.9)	<0.001
	non-users	90.8 (85.3-95.0)	90.8 (86.3-95.2)	0.83	90.6 (86.6-95.3)	0.69	89.4 (85.2-93.8)	<0.001	90.4 (86.5-93.8)	0.07
BUN (mg/dL)	users	57.8 (48.8-67.4)	57.2 (49.0-64.3)	0.13	58.9 (50.4-69.1)	0.42	54.2 (45.5-62.6)	0.004	51.2 (43.6-63.6)	0.002
	non-users	60.5 (51.8-70.3)	60.8 (52.0-71.8)	0.84	62.7 (50.8-71.3)	0.96	55.2 (46.5-64.9)	<0.001	54.4 (44.5-68.1)	<0.001
Cr (mg/dL)	users	9.26 (7.92-10.45)	9.23 (8.07-10.58)	0.66	8.99 (8.01- 9.98)	0.11	9.10 (7.94-10.12)	0.63	8.63 (7.52-10.25)	0.003
	non- users	8.97 (7.26-10.57)	8.77 (7.23-10.49)	0.20	8.94 (7.03-10.48)	0.23	8.56 (7.14-10.51)	0.54	8.45 (6.64-10.29)	<0.001
CRP (mg/dL)	users	0.16 (0.08-0.61)	0.15 (0.07-0.42)	0.54	0.14 (0.06-0.39)	0.79	0.20 (0.06-0.52)	0.29	0.20 (0.06-0.50)	0.55
	non- users	0.20 (0.08-0.49)	0.15 (0.06-0.49)	0.64	0.13 (0.05-0.45)	0.89	0.14 (0.05-0.47)	0.03	0.24 (0.08-0.61)	0.96

Wilcoxon signed rank test were used to compare the difference between 0 month and each month in a same group. DW, Dry weight; TG, triglycerides; TP, Total Protein; nPCR, normalized protein catabolism rate; BUN, blood urea nitrogen; Cr, creatinine; CRP, c-reactive protein; GNRI, geriatric nutritional risk index; aMedian (interquartile range)

Supplementary Table S2. Comparison of the decrease in parameters between cafeteria users and non-cafeteria users.

	1 month after			4 months after			7 months after			10 months after		
	users	non-users	*P*	users	non-users	*P*	users	non-users	*P*	users	non-users	*P*
ΔDW (kg)	0 (0-0)	0 (0-0)	0.42	0 (Δ0.5-0.5)	0 (Δ0.3-0.5)	0.22	Δ0.3 (Δ1.3-0.2)	0 (Δ0.5-1.0)	0.008	Δ0.5 (Δ2-0)	0 (Δ1-1.5)	<0.001
DW	0	0	0.46	0	0	0.24	Δ0.8	0	0.007	Δ1.2	0	<0.001

reduction rate (%)	(0-0)	(0-0)		(Δ0.9-0.8)	(Δ0.5-1.2)		(Δ2.7-0.4)	(Δ1.5-1.7)		(Δ4.2-0)	(Δ2.0-2.4)	
ΔTG (mg/dL)	Δ3 (Δ20-7)	1 (Δ12-16)	0.015	Δ9 (Δ25-6)	3 (Δ16-23)	0.019	Δ3 (Δ27-14)	2 (Δ11-24)	0.038	Δ3 (Δ25-16)	5 (Δ8-29)	0.039
ΔTP (g/dL)	0 (Δ0.2-0.1)	0 (Δ0.1-0.2)	0.12	Δ0.1 (Δ0.3-0.2)	0 (Δ0.2-0.2)	0.11	Δ0.2 (Δ0.3-0)	Δ0.1 (Δ0.3-0.1)	0.10	Δ0.1 (Δ0.3-0)	0 (Δ0.2-0.3)	0.006
ΔnPCR (g/kg/day)	0 (Δ0.1-0.1)	0.0 (0-0.1)	0.01	0.0 (Δ0.1-0.1)	0.1 (Δ0.1-0.1)	0.24	Δ0.0 (Δ0.1-0.1)	Δ0.0 (Δ0.1-0.1)	0.45	Δ0.0 (Δ0.1-0.1)	Δ0.0 (Δ0.1-0.1)	0.51
ΔGNRI	Δ0.3 (Δ1.5-1.5)	0 (Δ1.5-1.5)	0.41	Δ1.1 (Δ2.2-1.5)	0 (Δ2.5-1.9)	0.22	Δ1.5 (Δ3.8-0.2)	Δ1.5 (Δ4.2-1.4)	0.27	Δ1.5 (Δ4.9-1.2)	Δ0.9 (Δ3.5-1.8)	0.027
ΔBUN (mg/dL)	Δ1.2 (Δ8.2-5.1)	Δ0.3 (Δ8.0-6.6)	0.42	1.2 (Δ10.4-9.1)	Δ1.8 (Δ8.7-7.5)	0.63	Δ3.8 (Δ13.5-4.4)	Δ5.6 (Δ14.5-2.8)	0.43	Δ3.5 (Δ13.4-3.8)	Δ4.9 (Δ15.7-4.0)	0.63
ΔCr (mg/dL)	Δ0.1 (Δ0.3-0.4)	Δ0.1 (Δ0.4-0.3)	0.73	Δ0.2 (Δ0.9-0.3)	Δ0.1 (Δ0.8-0.5)	0.58	Δ0.1 (Δ0.9-0.7)	Δ0.2 (Δ0.7-0.6)	0.96	Δ0.4 (Δ1.4-0.5)	Δ0.5 (Δ1.3-0.4)	0.94
ΔCRP (mg/dL)	Δ0.01 (Δ0.1-0.1)	0 (Δ0.1-0.1)	0.46	0 (Δ0.1-0.1)	0 (Δ0.1-0)	0.98	0 (Δ0.1-0.2)	Δ0.02 (Δ0.2-0.0)	0.044	0 (Δ0.1-0.1)	0 (Δ0.1-0.2)	0.73

Wilcoxon sum test were used to compare the two groups. The decrease in each parameter was calculated by subtracting the value obtained in December 2020, which is the baseline, from the parameter in each month's dry weight measurement and blood examination. DW, Dry weight; TG, triglycerides; TP, Total Protein; nPCR, normalized protein catabolism rate; GNRI, geriatric nutritional risk index; BUN, blood urea nitrogen; Cr, creatinine; CRP, c-reactive protein; aMedian (interquartile range)
